# Influence of seasons on the management and outcomes acute myocardial infarction: An 18‐year US study

**DOI:** 10.1002/clc.23428

**Published:** 2020-08-06

**Authors:** Saraschandra Vallabhajosyula, Sri Harsha Patlolla, Wisit Cheungpasitporn, David R. Holmes, Bernard J. Gersh

**Affiliations:** ^1^ Department of Cardiovascular Medicine Mayo Clinic Rochester Minnesota USA; ^2^ Division of Pulmonary and Critical Care Medicine, Department of Medicine Mayo Clinic Rochester Minnesota USA; ^3^ Center for Clinical and Translational Science Mayo Clinic Graduate School of Biomedical Sciences Rochester Minnesota USA; ^4^ Section of Interventional Cardiology, Division of Cardiovascular Medicine, Department of Medicine Emory University School of Medicine Atlanta Georgia USA; ^5^ Department of Cardiovascular Surgery Mayo Clinic Rochester Minnesota USA; ^6^ Division of Nephrology, Department of Medicine University of Mississippi School of Medicine Jackson Mississippi USA

**Keywords:** acute myocardial infarction, healthcare disparities, outcomes research, season, winter

## Abstract

**Background:**

There are limited data on the seasonal variation in acute myocardial infarction (AMI) in the contemporary literature.

**Hypothesis:**

There would be decrease in the seasonal variation in the management and outcomes of AMI.

**Methods:**

Adult (>18 years) AMI admissions were identified using the National Inpatient Sample (2000‐2017). Seasons were classified as spring, summer, fall, and winter. Outcomes of interest included prevalence, in‐hospital mortality, use of coronary angiography, and percutaneous coronary intervention (PCI). Subgroup analyses for type of AMI and patient characteristics were performed.

**Results:**

Of the 10 880 856 AMI admissions, 24.3%, 22.9%, 22.2%, and 24.2% were admitted in spring, summer, fall, and winter, respectively. The four cohorts had comparable age, sex, race, and comorbidities distribution. Rates of coronary angiography and PCI were slightly but significantly lower in winter (62.6% and 40.7%) in comparison to the other seasons (64‐65% and 42‐43%, respectively) (*P* < .001). Compared to spring, winter admissions had higher in‐hospital mortality (adjusted odds ratio [aOR]: 1.07; 95% confidence interval [CI]: 1.06‐1.08), whereas summer (aOR 0.97; 95% CI 0.96‐0.98) and fall (aOR 0.98; 95% CI 0.97‐0.99) had slightly lower in‐hospital mortality (*P* < .001). ST‐segment elevation (10.0% vs 9.1%; aOR 1.07; 95% CI 1.06‐1.08) and non‐ST‐segment elevation (4.7% vs 4.2%; aOR 1.07; 95% CI 1.06‐1.09) AMI admissions in winter had higher in‐hospital mortality compared to spring (*P* < .001). The primary results were consistent when stratified by age, sex, race, geographic region, and admission year.

**Conclusions:**

Compared to other seasons, winter admission was associated with higher in‐hospital mortality in AMI in the United States.

**Abbreviations**
AMI: acute myocardial infarction
CI: confidence interval
GWTG‐CAD: Get With The Guideline‐Coronary Artery Disease
HCUP: Healthcare Cost and Utilization Project
ICD‐10CM: International Classification of Diseases‐10 Clinical Modification
ICD‐9CM: International Classification of Diseases‐9 Clinical Modification
NIS: National/Nationwide Inpatient Sample
NSTEMI: non‐ST‐segment elevation myocardial infarction
OR: odds ratio
PCI: percutaneous coronary intervention
STEMI: ST‐segment elevation myocardial infarction

## 1 INTRODUCTION

Studies evaluating the chronobiology of acute myocardial infarction (AMI) have reported a circadian and seasonal periodicity for the incidence of AMI.^1^, ^2^, ^3^, ^4^, ^5^, ^6^ Reports from large national registries have shown seasonal variations in AMI‐related mortality with the majority reporting increased incidence and in‐hospital mortality during the winter months compared to other seasons.^4^, ^5^, ^6^ Subsequent studies have evaluated the impact of daily weather and environmental conditions in well‐defined geographical areas further establishing the role of weather as a potential trigger for cardiovascular diseases.^7^, ^8^ However, only a selected few studies evaluated the seasonal association of AMI stratified by type of AMI (ST‐segment elevation myocardial infarction [STEMI] vs non‐ST‐segment elevation myocardial infarction [NSTEMI]).^9^, ^10^ Furthermore, several reports have shown that similarities exist between seasonal patterns of AMI and influenza infection.^11^, ^12^ Therefore through this study, we sought to assess the seasonal variations in clinical outcomes of AMI using an extensive national database over 18 years while comparing these differences in STEMI and NSTEMI populations. We hypothesized that with advances in healthcare deliveries there would be decrease in the seasonal variation in the management and outcomes of AMI.

## 2 MATERIAL AND METHODS

### 2.1 Study population, variables, and outcomes

The National (Nationwide) Inpatient Sample (NIS) is the largest all‐payer database of hospital inpatient stays in the United States. NIS contains discharge data from a 20% stratified sample of community hospitals and is a part of the Healthcare Quality and Utilization Project (HCUP), sponsored by the Agency for Healthcare Research and Quality.^13^ Information regarding each discharge includes patient demographics, primary payer, hospital characteristics, principal diagnosis, up to 24 secondary diagnoses, and procedural diagnoses. The HCUP‐NIS does not capture individual patients but captures all information for a given admission. Institutional Review Board approval was not sought due to the publicly available nature of this de‐identified database. These data are available to other authors via the HCUP‐NIS database with the Agency for Healthcare Research and Quality.

Using the HCUP‐NIS data from 2000 to 2017, a retrospective cohort study of adult admissions (>18 years) with AMI in the primary diagnosis field (International Classification of Diseases 9.0 Clinical Modification [ICD‐9CM] 410.x and ICD‐10CM I21.x‐22.x) were identified. Similar to prior literature, we defined the seasons based on the meteorological classification of the Northern Hemisphere as—Spring (March‐May), Summer (June‐August), Fall (September‐November), and Winter (December‐February).^14^ We excluded admissions that did not have information on admission month. The Deyo's modification of the Charlson Comorbidity Index was used to identify the burden of comorbid diseases (Table S1).^15^ Demographic characteristics, hospital characteristics, acute organ failure, mechanical circulatory support, cardiac procedures, and noncardiac organ support use were identified for all admissions using previously used methodologies from our group.^16^, ^17^, ^18^, ^19^, ^20^, ^21^, ^22^, ^23^, ^24^, ^25^, ^26^, ^27^, ^28^, ^29^, ^30^, ^31^, ^32^, ^33^, ^34^, ^35^, ^36^, ^37^, ^38^, ^39^, ^40^, ^41^, ^42^, ^43^, ^44^, ^45^, ^46^, ^47^ The four geographic regions included the Northeast, Midwest, South, and West as classified by the HCUP‐NIS.^35^ Similar to prior literature, we defined early coronary angiography as that performed on the day of hospital admission (day 0).^31^, ^42^, ^43^ We identified timing of coronary angiography and percutaneous coronary intervention (PCI) relative to the day of admission.^18^, ^31^, ^41^, ^42^

The primary outcome was the seasonal variation in the prevalence of AMI and the in‐hospital mortality in admissions with AMI. The secondary outcomes included receipt of coronary angiography, PCI and mechanical circulatory support, hospital length of stay, hospitalization costs, and discharge disposition. Stratified analyses were performed for type of AMI (STEMI vs NSTEMI) and patient characteristics (age, sex, race, tertile of study period, and geographic region).

### 2.2 Statistical analysis

As recommended by HCUP‐NIS, survey procedures using discharge weights provided with HCUP‐NIS database were used to generate national estimates.^48^ Using the trend weights provided by the HCUP‐NIS, samples from 2000 to 2011 were reweighted to adjust for the 2012 HCUP‐NIS redesign.^48^ Chi‐square and *t* tests were used to compare categorical and continuous variables, respectively. Multivariable logistic regression was used to analyze trends over time (referent year 2000). Univariable analysis for trends and outcomes was performed and was represented as odds ratio (OR) with 95% confidence interval (CI). Multivariable logistic regression analysis incorporating age, sex, race, primary payer status, weekend admission, socioeconomic stratum, hospital characteristics, comorbidities, organ failure, AMI‐type, cardiac procedures, and noncardiac procedures was performed for assessing temporal trends of prevalence and in‐hospital mortality. To confirm the results of the primary analysis, multiple subgroup analyses stratifying by age, sex, race, tertiles of study period, type of AMI, and geographic region were performed. For the multivariable modeling, regression analysis with purposeful selection of statistically (liberal threshold of *P* < .20 in univariate analysis) and clinically relevant variables was conducted. Two‐tailed *P* < .05 was considered statistically significant. All statistical analyses were performed using SPSS v25.0 (IBM Corp, Armonk, New York).

Best practices relating to the use of the HCUP‐NIS database, such as not assessing individual hospital‐level volumes (due to changes to sampling design detailed above), treating each entry as an “admission” as opposed to individual patients, restricting the study details to inpatient factors since the HCUP‐NIS does not include outpatient data, and limiting administrative codes to those previously validated and used for similar studies, were adhered to during data analysis.^48^

## 3 RESULTS

**FIGURE 1:**
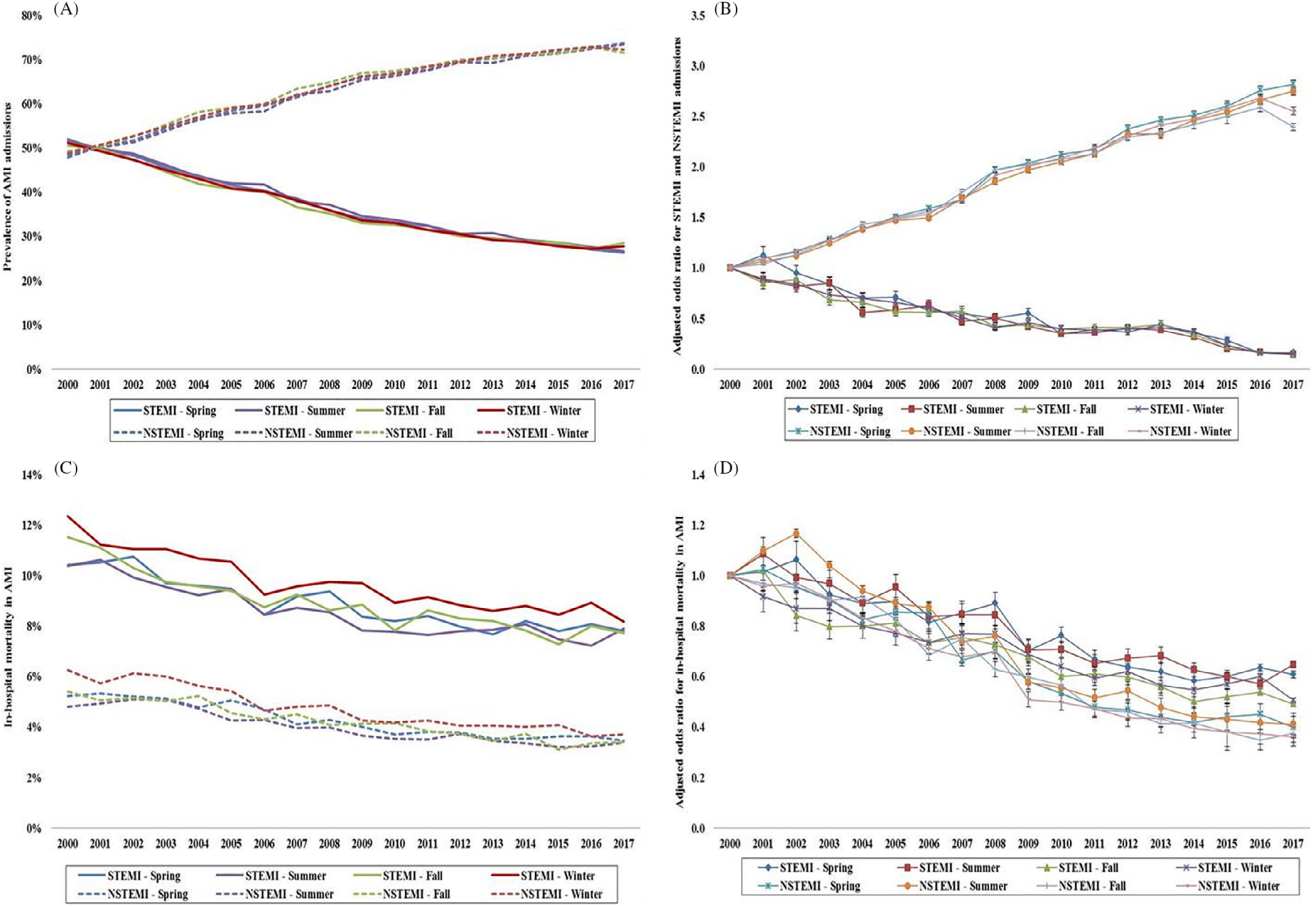
Trends in the prevalence and in‐hospital mortality in AMI admissions stratified by type of AMI. A, Unadjusted temporal trends of the proportion of AMI admissions stratified by type of AMI during spring, summer, fall, and winter (*P* < .001 for trend over time). B, Adjusted odds ratio for STEMI and NSTEMI weekend admissions by year (with 2000 as the referent); adjusted for age, sex, race, comorbidity, primary payer, socioeconomic status, STEMI location, hospital region, hospital location and teaching status, and hospital bed‐size (*P* < .001 for trend over time). C, Unadjusted in‐hospital mortality in AMI admissions stratified by type of AMI during spring, summer, fall, and winter (*P* < .001 for trend over time). D, Adjusted odds ratio for in‐hospital mortality by year (with 2000 as the referent) in AMI admissions stratified by type of AMI and weekend vs weekday admission; adjusted for age, sex, race, comorbidity, primary payer, hospital region, hospital location and teaching status, hospital bed‐size, weekend admission, multiorgan failure, cardiogenic shock, cardiac arrest, coronary angiography, PCI, pulmonary artery catheterization, mechanical circulatory support, invasive mechanical ventilation, and acute hemodialysis (*P* < .001 for trend over time). AMI, acute myocardial infarction; NSTEMI, non‐ST‐segment elevation myocardial infarction; STEMI, ST‐segment elevation myocardial infarction

In the period from 1 January 2000 to 31 December 2017, there were 11 622 528 admissions for AMI, of which, 741 672 did not have data on the month of admission and were excluded. Among the final cohort of 10 880 856 (93.6%) admissions, 2 826 906 (24.3%), 2 660 729 (22.9%), 2 577 885 (22.2%), and 2 815 336 (24.2%), respectively were admitted in spring, summer, fall, and winter (Figure S1). The 18‐year temporal trends of AMI admissions showed a consistent increase in NSTEMI admissions with a concomitant decrease in STEMI rates during this study period without significant differences between the seasons (Figure 1A,B). The four cohorts had comparable distribution of STEMI vs NSTEMI, age, sex, race, insurance, socioeconomic status, and comorbidity (Table 1). NSTEMI admissions comprised 62% to 63% of all admissions across the four seasons during the study period. There were no clinically meaningful differences in the hospital characteristics (location, teaching status, bed‐size, and region) between the four cohorts (Table 1). Cardiac arrest, cardiogenic shock, and multiorgan failure were noted in about 5%, 5%, and 9%, respectively across the seasons and were comparable across the four cohorts.

**TABLE 1:** Baseline and clinical characteristics of AMI admissions stratified by seasons

Characteristic	Spring (N = 2 826 906)	Summer (N = 2 660 729)	Fall (N = 2 577 885)	Winter (N = 2 815 336)	*P*
Type of AMI					<.001
STEMI	37.1	37.4	37.1	37.1	
NSTEMI	62.9	62.6	62.9	62.9	
Age (y)	67.4 ± 14.2	67.0 ± 14.3	67.5 ± 14.2	67.9 ± 14.2	<.001
Female sex	39.5	39.5	40.0	39.9	<.001
Race					<.00
White	63.1	62.8	62.4	63.1	
Black	7.9	8.0	7.9	7.8	1
Others^a^	29.0	29.2	29.7	29.1	
Primary payer					<.001
Medicare	57.2	56.2	57.4	58.2	
Medicaid	6.3	6.4	6.2	6.1	
Private	28.3	28.8	28.1	27.6	
Others^b^	8.1	8.5	8.4	8.1	
Quartile of median household income for zip code					<.001
0‐25th	24.6	24.5	24.1	24.4	
26th‐50th	26.9	26.9	26.9	26.9	
51st‐75th	24.2	24.3	24.3	24.3	
75th‐100th	24.2	24.3	24.8	24.4	
Charlson Comorbidity Index					<.001
0–3	38.7	40.2	36.4	36.6	
4–6	43.8	43.1	44.8	44.9	
≥7	17.5	16.7	18.7	18.5	
Prior coronary artery bypass grafting	7.8	7.7	7.8	7.9	<.001
Hospital teaching status and location					<.001
Rural	11.6	11.5	11.6	11.6	
Urban nonteaching	37.4	37.3	37.9	38.2	
Urban teaching	51.0	51.2	50.5	50.2	
Hospital bed‐size					<.001
Small	11.9	11.6	11.5	11.8	
Medium	26.1	25.9	25.8	26.1	
Large	62.0	62.4	62.7	62.1	
Hospital region					<.001
Northeast	20.8	20.9	21.0	21.0	
Midwest	24.6	24.7	24.5	23.9	
South	36.1	36.1	35.9	36.2	
West	18.4	18.3	18.5	18.9	
Tertile of admission years					<.001
2000‐2005	34.9	34.7	36.9	36.2	
2006‐2011	30.1	29.9	31.3	30.4	
2012‐2017	35.0	35.4	31.8	33.4	
Weekend admission	25.8	25.9	25.8	25.7	<.001
Fibrinolytic therapy (STEMI only)	4.7	4.6	4.5	4.7	<.001
Coronary artery bypass grafting	9.4	9.1	9.2	9.2	<.001
Cardiac arrest	4.9	5.0	5.1	5.1	<.001
Cardiogenic shock	4.9	4.8	4.8	4.9	<.001
Multiorgan failure	9.4	9.2	9.3	9.8	<.001
Respiratory infections					
Influenza	0.1	0.0	0.0	0.4	<.001
Pneumonia	6.9	5.6	6.3	8.0	<.001
Fibrinolysis	2.2	2.2	2.1	2.2	<.001
Pulmonary artery catheterization	1.1	1.0	1.0	1.1	<.001
Invasive mechanical ventilation	5.9	5.7	5.8	6.2	<.001
Acute hemodialysis	0.5	0.5	0.6	0.6	<.001

*Note:* Represented as percentage or mean ± SD.

Abbreviations: AMI, acute myocardial infarction; NSTEMI, non‐ST‐segment‐elevation myocardial infarction; STEMI, ST‐segment‐elevation myocardial infarction.

a Hispanic, Asian or Pacific Islander, Native American, others.

b Self‐pay, no charge, others.

**FIGURE 2:**
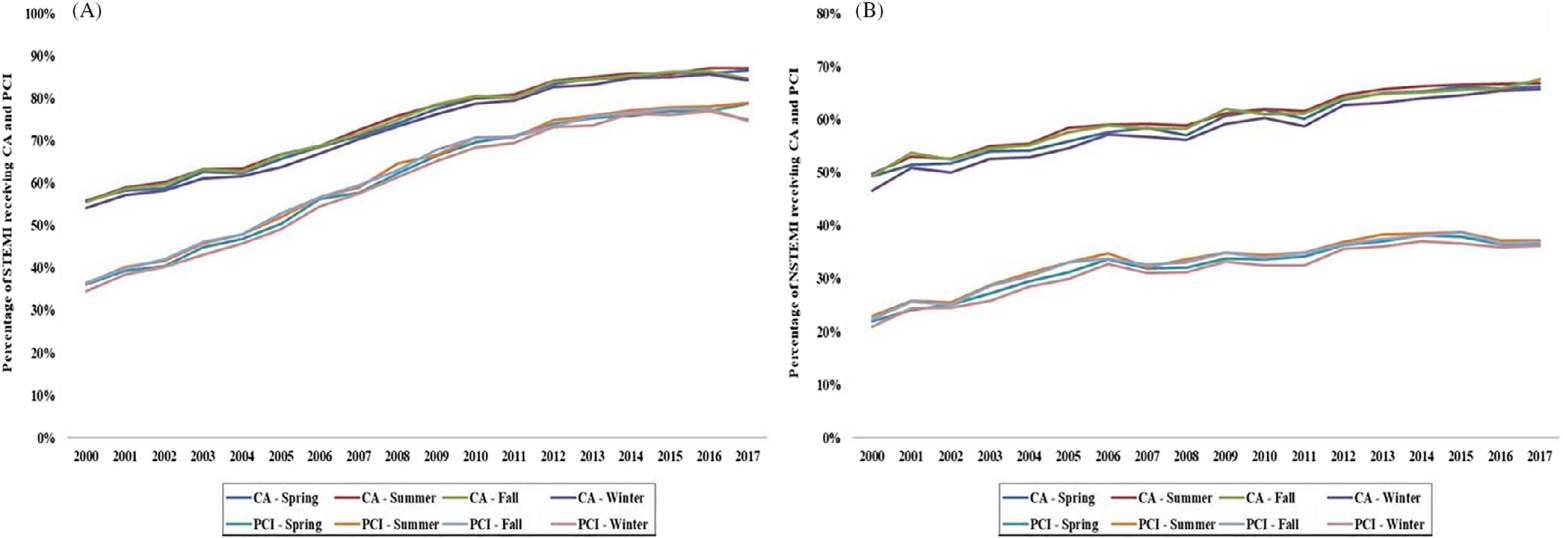
Temporal trends in the use of CA and PCI stratified by type of AMI. Eighteen‐year temporal trends in the use of CA and PCI in STEMI (A) and NSTEMI (B); all *P* < .001 for trend over time. AMI, acute myocardial infarction; CA, coronary angiography; NSTEMI, non‐ST‐segment elevation myocardial infarction; PCI, percutaneous coronary intervention; STEMI, ST‐segment elevation myocardial infarction

Rates of coronary angiography and PCI were slightly but significantly lower in winter (62.6% and 40.7%) in comparison to the other three seasons (64%‐65% and 42%‐43%, respectively) (*P* < .001) (Table 2). During the 18‐year study period, the STEMI admissions underwent comparable rates of coronary angiography and PCI (Figure 2A,C), whereas the NSTEMI admissions in winter received consistently lower use of both procedures compared to other seasons (Figure 2B,D). The use of mechanical circulatory support was comparable during the seasons (Table 2). Hospital costs, length of hospital stay, and discharge dispositions were similar across seasons (Table 2).

**TABLE 2:** Clinical outcomes of AMI admissions stratified by seasons

Characteristic	Spring (N = 2 826 906)	Summer (N = 2 660 729)	Fall (N = 2 577 885)	Winter (N = 2 815 336)	*P*
Coronary angiography	64.0	65.0	64.1	62.6	<.001
Percutaneous coronary intervention	42.0	43.0	42.1	40.7	<.001
Mechanical circulatory support	4.9	4.7	4.8	4.8	<.001
In‐hospital mortality	6.0	5.8	6.1	6.7	<.001
Length of stay (days)	5.1 ± 5.8	4.9 ± 5.6	5.0 ± 5.9	5.2 ± 6.0	<.001
Hospitalization costs (×1000 USD)	60 ± 79	60 ± 77	60 ± 79	60 ± 81	<.001
Discharge disposition					<.001
Home	62.9	64.0	63.2	62.1	
Transfer	12.4	12.2	12.4	12.4	
Skilled nursing facility	13.2	12.8	13.2	14.0	
Home with HHC	10.6	10.1	10.3	10.7	
Against medical advice	0.9	0.9	0.9	0.8	

*Note:* Represented as percentage or mean ± SD.

Abbreviations: AMI, acute myocardial infarction; HHC, home healthcare; USD, United States Dollars.

The AMI admissions in winter had slightly higher in‐hospital mortality 6.7% compared to other seasons 5.8% to 6.1% (*P* < .001). During this 18‐year period, winter admissions had consistently higher in‐hospital mortality compared to other seasons in both STEMI and NSTEMI subgroups (Figure 1C,D). In a multivariable analysis, with spring as the referent category, AMI admissions in winter had slightly higher adjusted in‐hospital mortality (OR 1.07; 95% CI 1.06‐1.08), whereas those in summer (OR 0.97; 95% CI 0.97‐0.98) and fall (OR 0.98; 95% CI 0.98‐0.99) had slightly lower in‐hospital mortality (*P* < .001) (Table S2). These results were largely consistent in the subgroup analyses. Winter AMI admissions had higher in‐hospital mortality in both STEMI and NSTEMI admissions (Table 3). When stratifying by age, sex, and race, compared to spring, summer admissions had lower in‐hospital mortality whereas winter admissions had higher in‐hospital mortality (Table 3). When stratified by tertiles of study period, these differences became less pronounced over time (Table 3). The primary outcome did not differ between the four geographic regions (Table 3).

**TABLE 3:** In‐hospital mortality in AMI admissions stratified by patient characteristics

Patient characteristics^a^	Odds ratio	95% confidence interval		*P*
		Lower Limit	Upper Limit	
Type of AMI				
STEMI				
Spring	Reference category			
Summer	0.97	0.97	0.98	<.001
Fall	0.98	0.98	0.99	<.001
Winter	1.07	1.06	1.08	<.001
NSTEMI				
Spring	Reference category			
Summer	0.98	0.97	0.99	.003
Fall	1.00	0.99	1.01	.64
Winter	1.07	1.06	1.09	<.001
Age group				
≤75 years				
Spring	Reference category			
Summer	0.98	0.96	0.99	<.001
Fall	0.97	0.95	0.98	<.001
Winter	1.07	1.06	1.08	<.001
>75 years				
Spring	Reference category			
Summer	0.98	0.96	0.99	<.001
Fall	1.00	0.99	1.01	.88
Winter	1.07	1.06	1.08	<.001
Sex				
Male				
Spring	Reference category			
Summer	0.97	0.96	0.98	<.001
Fall	0.99	0.98	1.01	.18
Winter	1.08	1.06	1.09	<.001
Female				
Spring	Reference category			
Summer	0.98	0.97	0.99	.003
Fall	0.98	0.97	0.99	<.001
Winter	1.06	1.05	1.08	<.001
Race				
White				
Spring	Reference category			
Summer	0.98	0.97	0.99	<.001
Fall	0.99	0.98	0.99	.04
Winter	1.07	1.05	1.08	<.001
Non‐White^b^				
Spring	Reference category			
Summer	0.97	0.96	0.98	<.001
Fall	0.98	0.96	0.99	.003
Winter	1.08	1.06	1.09	<.001
Tertiles of study period				
2000‐2005				
Spring	Reference category			
Summer	0.99	0.99	1.01	.80
Fall	1.02	1.00	1.03	.01
Winter	1.10	1.08	1.11	<.001
2006‐2011				
Spring	Reference category			
Summer	0.95	0.94	0.97	<.001
Fall	0.99	0.97	1.01	.22
Winter	1.06	1.04	1.08	<.001
2012‐2017				
Spring	Reference category			
Summer	0.97	0.95	0.98	<.001
Fall	0.94	0.92	0.96	<.001
Winter	1.04	1.03	1.06	<.001
Geographic region				
Northeast				
Spring	Reference category			
Summer	0.97	0.95	0.98	<.001
Fall	0.98	0.96	0.99	.02
Winter	1.08	1.06	1.10	<.001
Midwest				
Spring	Reference category			
Summer	0.98	0.96	0.99	.007
Fall	0.98	0.97	1.00	.05
Winter	1.08	1.06	1.09	<.001
South				
Spring	Reference category			
Summer	0.98	0.96	0.99	.001
Fall	0.98	0.97	0.99	.02
Winter	1.07	1.05	1.09	<.001
West				
Spring	Reference category			
Summer	0.99	0.97	1.01	0.16
Fall	1.00	0.98	1.02	0.71
Winter	1.05	1.03	1.07	<.001

Abbreviations: AMIE, acute myocardial infarction; NSTEMI, non‐ST‐segment‐elevation myocardial infarction; STEMI, ST‐segment‐elevation myocardial infarction.

a Each subgroup was adjusted for age, sex, race, insurance status, socioeconomic stratum, hospital characteristics, comorbidities, year of admission, weekend admission, cardiogenic shock, cardiac arrest, multiorgan failure, respiratory infections, coronary angiography, percutaneous coronary intervention, pulmonary artery catheterization, mechanical circulatory support, and invasive mechanical ventilation.

b Black, Hispanic, Asian, Native American, others.

## 4 DISCUSSION

In the largest study evaluating seasonal effect on the management and outcomes of nearly 11 million AMI admissions, we noted winter admissions with AMI to have higher in‐hospital mortality which was more pronounced in the NSTEMI population. Despite comparable baseline characteristics and acuity of illness, the AMI admissions in winter had slightly, but statistically significant, lower rates of coronary angiography and PCI use. These disparities were persistent during the 18‐year study period, however, were less pronounced over time. These results were consistent in both STEMI and NSTEMI and across all patient demographics.

To date, in addition to several small cohort‐studies,^49^, ^50^, ^51^ reports from large national databases of various countries have demonstrated a seasonal pattern to the incidence of AMI.^4^, ^5^, ^6^ In the United States, two different studies from the National Registry of Myocardial Infarction have reported a higher number of AMI cases in winter and a significantly lower number in summers.^4^, ^5^ However, these data was obtained from about 15% of all acute medical/surgical hospitals in the United States, which limits the generalizability.^5^ Subsequently, Patel et al evaluated seasonal variations in AMI incidence using the HCUP‐NIS database from 2000 to 2011 and identified a marked increase in AMI hospitalizations during winter among the elderly population but found no such association in those younger than 65 years.^52^ More recently, data from Get With The Guideline‐Coronary Artery Disease (GWTG‐CAD) Program, which included a significant proportion of patients with AMI in the United States from 2003 to 2008, showed evidence of a seasonal variation in the incidence of AMI. However, the seasonal pattern was limited only to those with NSTEMI and was not significant in STEMI patients. Besides, they also reported that seasonal variation with winter predominance was identified only in the warmer states of the country.^9^ These reports suggest that seasonal variation in AMI incidence may not be as uniform across the nation as previously believed. In comparison to these studies, our data did not show differences in the overall prevalence of AMI when evaluated by seasons. Due to the differences in the inclusion criteria (ie, AMI vs all acute coronary syndromes including unstable angina), the nationally representative nature of this study, the large sample size of our population and the evolution of medical therapy for AMI might explain some of these differences.

These differences may partly be explained by the inclusion criteria for these cohorts and patient selection. Our study represents the largest national cohort and spans over a significantly longer duration. Additionally, while we identified patients based on ICD codes, studies from NRMI and GWTG‐CAD used clinical findings in conjunction with ICD codes. Furthermore, it is important to consider the inclusion of all acute coronary syndrome patients (ie, including unstable angina) vs only those with a true AMI. Moreover, another population‐based study using clinical findings to identify AMI reported a lack of seasonal variation in incident AMI cases.^8^ Discrepancies with results from other countries could potentially be explained by the fact that the United States has a diverse climate across the nation independent of seasons, unlike other countries which have reported a seasonal variation. In this regard, there appears to be no seasonal variation based upon geographic region. Another interesting trend identified in our study was the consistent increase in NSTEMI admissions over the 18 years. Contemporary evidence has shown a decline in the incidence of all types of AMI.^53^, ^54^, ^55^ Improvements in evidence‐based management strategies, greater emphasis on primary prevention, and increased use of cardioprotective medications and prior coronary revascularization are the reasons for an overall decline in AMI incidence.^53^ However, a simultaneous increase in the usage and sensitivity of cardiac biomarkers, specifically for NSTEMI, could potentially be one of the many reasons for the increasing trend in NSTEMI incidence.^54^, ^55^ Given the higher rate of NSTEMI in the elderly, an increase in the elderly population over the last decade could also explain this trend.^9^, ^55^ In order to ensure we are only capturing a type‐1 NSTEMI, this study only included admissions with a primary diagnosis of NSTEMI.

Despite the lack of seasonal association in the incidence of AMI, we did find small, but significant, variations in the in‐hospital mortality across various seasons. Both unadjusted and adjusted analyses showed increased in‐hospital mortality during winters and the lowest in summers in both STEMI and NSTEMI admissions, as well as the entire AMI cohort. Spencer et al reported higher in‐hospital case‐fatality rates for AMI during winter and the lowest in spring.^5^ Another study from Canada identified a seasonal variation in mortality of nearly 10%, with the highest number of AMI deaths in winter and lowest in summer.^56^ Similar findings were also reported from studies in German and Japanese populations.^6^, ^50^ In contrast, Nagarajan et al found no specific differences in in‐hospital mortality across seasons using the Get With The Guidelines registry from the United States.^9^ However, when stratified into STEMI and NSTEMI, they found higher mortality among the STEMI admissions during the fall season.^9^ Although inconclusive, these seasonal variations in AMI mortality have been attributed to factors such as intolerance to low temperatures among the elderly who constitute a significant part of the AMI population and others such as hemodynamic and physiologic changes associated with cooler temperatures.^5^, ^50^, ^52^ The association between the lower rates of coronary angiography and PCI for winter admissions, as identified in our study, is of potential concern. It is possible that weather conditions might have impacted total ischemic time in the winter; however, our study did not show any differences across all four geographic regions. Lower use of angiography in winter may be postulated to be due to higher rates of NSTEMI or type‐2 AMI since these patients may typically be admitted with respiratory illnesses. Respiratory illnesses might be associated with higher in‐hospital mortality, as noted in this study, and may additionally serve as barriers to coronary angiography and PCI due to concerns for overall patient trajectory.^57^, ^58^

### 4.1 Limitations

This study has several limitations, despite the HCUP‐NIS database's attempts to mitigate potential errors by using internal and external quality control measures. The administrative codes for AMI have been previously validated that reduces the inherent errors in the study. Echocardiographic data, angiographic variables, and hemodynamic parameters were unavailable in this database which limits physiological assessments of disease severity. We are unable to assess further detailed metrics such as total ischemic time and door‐to‐balloon time. Important factors such as the delay in presentation from time of onset of AMI symptoms, timing of cardiogenic shock, cardiac arrest, and acute organ failure, reasons for not receiving aggressive medical care, and treatment‐limiting decisions of organ support could not be reliably identified in this database. It is possible that despite best attempts at controlling for confounders by a multivariate analysis, winter admission was a marker of greater illness severity due to residual confounding. Despite these limitations, this study addresses an important knowledge gap highlighting the national temporal evolution of the seasonal effect and the impact of concomitant influenza infection on AMI.

## 5 CONCLUSIONS

In this study of nearly 11 million AMI admissions, winter admission was associated with higher in‐hospital mortality during this 18‐year study period. Though concomitant respiratory infections may explain this mortality, further data on the seasonal differences in outcomes relating to weather and travel and delayed presentations are needed to help understand this phenomenon better.

## AUTHOR CONTRIBUTIONS

SV, SHP, WC did study design, literature review, statistical analysis. SV, SHP, WC did data management, data analysis, drafting manuscript. SV, SHP, WC, DRH, BJG: Access to data. DRH, BJG: Manuscript revision, intellectual revisions, mentorship. SV, SHP, WC, DRH, BJG. Final approval.

## DISCLOSURE OF INTERESTS

All authors have reported that they have no relationships relevant to the contents of this paper to disclose.

## Supporting information


**Figure S1**. Temporal trends of total AMI, STEMI, and NSTEMI admissions during the study period. Unadjusted temporal trends of total AMI, STEMI, and NSTEMI admissions during the 18‐year study period (*P* < .001 for trend over time). AMI, acute myocardial infarction; NSTEMI, non‐ST‐segment elevation myocardial infarction; STEMI, ST‐segment elevation myocardial infarction

**Table S1**. Administrative codes used for identification of diagnoses and procedures
**Table S2**. Multivariable regression for in‐hospital mortality
