# Supplementary figures and images for: Influence of seasons on the management and outcomes acute myocardial infarction: An 18‐year US study
Source: Clin Cardiol. 2020 Aug 6;43(10):1175–85. doi: 10.1002/clc.23428 (PMC7533976; doi:10.1002/clc.23428)

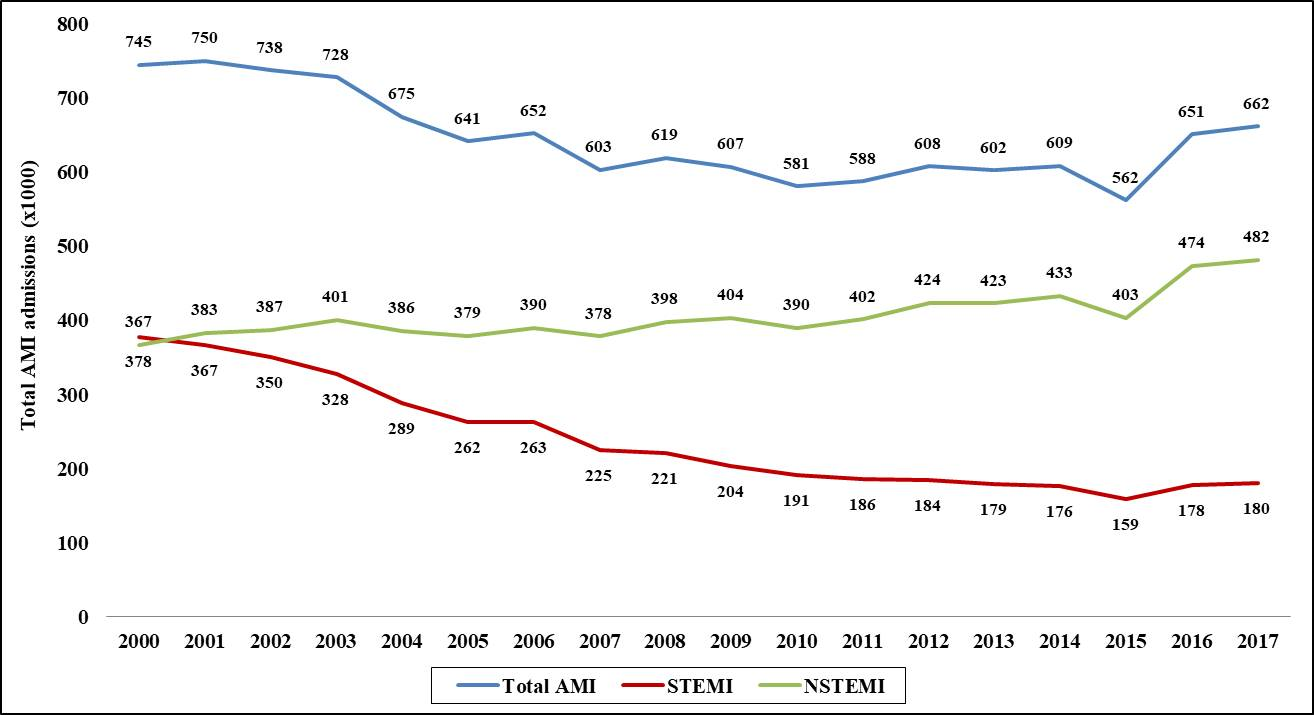

Supplement: Supplementary file 1 — Figure S1. Temporal trends of total AMI, STEMI, and NSTEMI admissions during the study period. Unadjusted temporal trends of total AMI, STEMI, and NSTEMI admissions during the 18‐year study period (P < .001 for trend over time). AMI, acute myocardial infarction; NSTEMI, non‐ST‐segment elevation myocardial infarction; STEMI, ST‐segment elevation myocardial infarction [file CLC-43-1175-s001.tif]
