# Supplementary material for: Influence of seasons on the management and outcomes acute myocardial infarction: An 18‐year US study
Source: Clin Cardiol. 2020 Aug 6;43(10):1175–85. doi: 10.1002/clc.23428 (PMC7533976; doi:10.1002/clc.23428)
Supplement: Supplementary file 2 — Table S1. Administrative codes used for identification of diagnoses and procedures Table S2. Multivariable regression for in‐hospital mortality [file CLC-43-1175-s002.docx]

**SUPPLEMENTARY MATERIAL**

**Influence of Seasons on the Management and Outcomes Acute Myocardial Infarction: An 18-Year United States Study**

Saraschandra Vallabhajosyula, MD MSc; Sri Harsha Patlolla, MBBS; Wisit Cheungpasitporn, MD; David R Holmes, Jr, MD; Bernard J Gersh, MBChB DPhil

**Supplementary Table 1. Administrative codes used for identification of diagnoses and procedures**

| **Comorbidity** | **International Classification of Diseases 9.0 Clinical Modification Codes** |
| --- | --- |
| Cardiac arrest | 427.5 |
| Coronary angiography | 37.22, 37.23, 88.53-88.56 |
| Percutaneous coronary intervention | 00.66, 36.01, 36.02, 36.05, 36.06, 36.07, 88.57 |
| Invasive hemodynamic assessment | 37.21, 37.23, 204 (Clinical Classification Software) |
| Mechanical circulatory support | 37.61, 37.68, 39.65 |
| Invasive mechanical ventilation | 96.7, 96.70, 96.71, 96.72 |
| Non-invasive mechanical ventilation | 93.90 |
| Hemodialysis | 39.95 |
| Hepatic failure | 570.0, 572.2, 573.3, 573.4 |
| Respiratory failure | 518.81, 518.82, 518.85, 786.09, 799.1, 96.7, 96.70, 96.71, 96.72 |
| Renal failure | 584, 584.5, 584.6, 584.7, 584.8, 584.9 |
| Hematologic failure | 286.6-286.9, 287.4, 287.5 |
| Neurological failure | 293, 293.0, 293.1, 293.8, 293.81-293.84, 293.89, 293.9, 348.1, 348.3, 348.30, 348.81, 348.39, 780.01, 780.09, 89.14 |

**Supplementary Table 2. Multivariable regression for in-hospital mortality**

| **Total cohort**  **(N = 10,880,856)** | | **Odds ratio** | **95% confidence interval** | | ***P*** |
| --- | --- | --- | --- | --- | --- |
|  |  |  | **Lower Limit** | **Upper Limit** |  |
| **Season** | **Spring** | Reference category | | | |
|  | **Summer** | 0.97 | 0.97 | 0.98 | <0.001 |
|  | **Fall** | 0.98 | 0.98 | 0.99 | <0.001 |
|  | **Winter** | 1.07 | 1.06 | 1.08 | <0.001 |
| **Age (years)** | **≤75 years** | Reference category | | | |
|  | **>75 years** | 2.16 | 2.14 | 2.17 | <0.001 |
| **Female sex** | | 1.15 | 1.15 | 1.16 | <0.001 |
| **Race** | **White** | Reference category | | | |
|  | **Black** | 0.85 | 0.83 | 0.86 | <0.001 |
|  | **Others** | 1.00 | 0.99 | 1.01 | 0.853 |
| **Primary payer** | **Medicare** | Reference category | | | |
|  | **Medicaid** | 0.80 | 0.78 | 0.81 | <0.001 |
|  | **Private** | 0.65 | 0.65 | 0.66 | <0.001 |
|  | **Others** | 0.93 | 0.92 | 0.95 | <0.001 |
| **Charlson comorbidity index** | **0-3** | Reference category | | | |
|  | **4-6** | 2.01 | 1.99 | 2.03 | <0.001 |
|  | **≥ 7** | 2.58 | 2.55 | 2.61 | <0.001 |
| **Quartile of median household**  **income for zip code** | **0-25^th^** | Reference category | | | |
|  | **26^th^-50^th^** | 0.99 | 0.98 | 1.00 | 0.003 |
|  | **51^st^-75^th^** | 0.94 | 0.93 | 0.95 | <0.001 |
|  | **75^th^-100^th^** | 0.92 | 0.91 | 0.92 | <0.001 |
| **Hospital teaching**  **status and location** | **Rural** | Reference category | | | |
|  | **Urban Non-Teaching** | 1.06 | 1.05 | 1.07 | <0.001 |
|  | **Urban Teaching** | 1.15 | 1.13 | 1.16 | <0.001 |
| **Hospital bed-size** | **Small** | Reference category | | | |
|  | **Medium** | 1.05 | 1.04 | 1.06 | <0.001 |
|  | **Large** | 1.14 | 1.13 | 1.15 | <0.001 |
| **Hospital region** | **Northeast** | Reference category | | | |
|  | **Midwest** | 1.00 | 0.99 | 1.01 | 0.676 |
|  | **South** | 1.10 | 1.09 | 1.11 | <0.001 |
|  | **West** | 0.87 | 0.86 | 0.88 | <0.001 |
| **Weekend admission** | | 1.02 | 1.02 | 1.03 | <0.001 |
| **Tertiles of admissions year** | **2000-2005** | Reference category | | | |
|  | **2006-2011** | 0.73 | 0.72 | 0.73 | <0.001 |
|  | **2012-2017** | 0.57 | 0.56 | 0.57 | <0.001 |
| **Type of AMI** | **STEMI** | Reference category | | | |
|  | **NSTEMI** | 0.43 | 0.43 | 0.44 | <0.001 |
| **Cardiogenic shock** | | 3.07 | 3.04 | 3.10 | <0.001 |
| **Cardiac arrest** | | 10.57 | 10.48 | 10.66 | <0.001 |
| **Multiorgan failure** | | 3.11 | 3.08 | 3.13 | <0.001 |
| **Influenza** | | 0.65 | 0.60 | 0.70 | <0.001 |
| **Pneumonia** | | 1.26 | 1.25 | 1.27 | <0.001 |
| **Coronary angiography** | | 0.33 | 0.33 | 0.33 | <0.001 |
| **Percutaneous coronary intervention** | | 0.51 | 0.50 | 0.51 | <0.001 |
| **Pulmonary artery catheterization** | | 1.15 | 1.13 | 1.18 | <0.001 |
| **Mechanical circulatory support** | | 1.69 | 1.67 | 1.71 | <0.001 |
| **Invasive mechanical ventilation** | | 3.06 | 3.03 | 3.08 | <0.001 |
| **Acute hemodialysis** | | 1.63 | 1.59 | 1.67 | <0.001 |

**Abbreviations:** AMI: acute myocardial infarction; NSTEMI: non-ST-segment-elevation myocardial infarction; STEMI: ST-segment-elevation myocardial infarction
